# Supplementary material for: Cross-Talk between CB1, AT1, AT2 and Mas Receptors Responsible for Blood Pressure Control in the Paraventricular Nucleus of Hypothalamus in Conscious Spontaneously Hypertensive Rats and Their Normotensive Controls
Source: Cells. 2022 May 4;11(9):1542. doi: 10.3390/cells11091542 (PMC9101384; doi:10.3390/cells11091542)
Supplement: Supplementary file 1 [file cells-11-01542-s001.zip › cells-1684925-supplementary.pdf]

SUPPLEMENTARY MATERIAL

Cross-talk between CB_1, AT_1, AT_2 and Mas receptors responsible for blood pressure control in the paraventricular nucleus of hypothalamus in conscious spontaneously hypertensive rats and their normotensive controls

Krzysztof Mińczuk, Eberhard Schlicker, Barbara Malinowska

CB_1-R

From the left (rows):
standard (1), **PVN** WKY (2-7), **PVN** SHR (8-13), **RVLM** WKY (14-20), **RVLM** SHR (21-26)

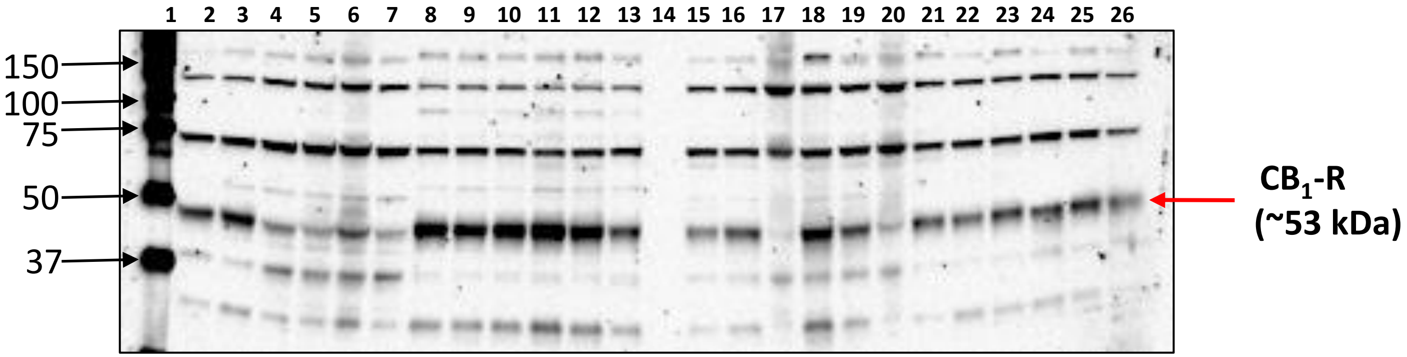

standard (1), **NTS** WKY (2-7), **NTS** SHR (8-13)

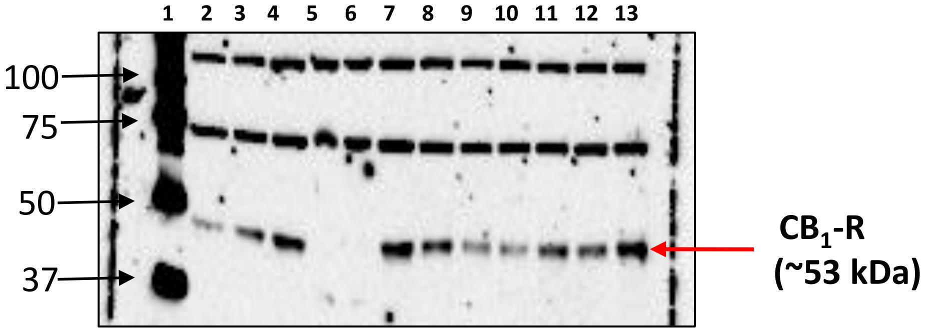

Figure S1a. Uncropped Western blots shown in Figure 7a.

AT_2-R

From the left (rows):
standard (1), **PVN** WKY (2-7), **PVN** SHR (8-13)

RVLM WKY (1-6), **RVLM** SHR (7-12)

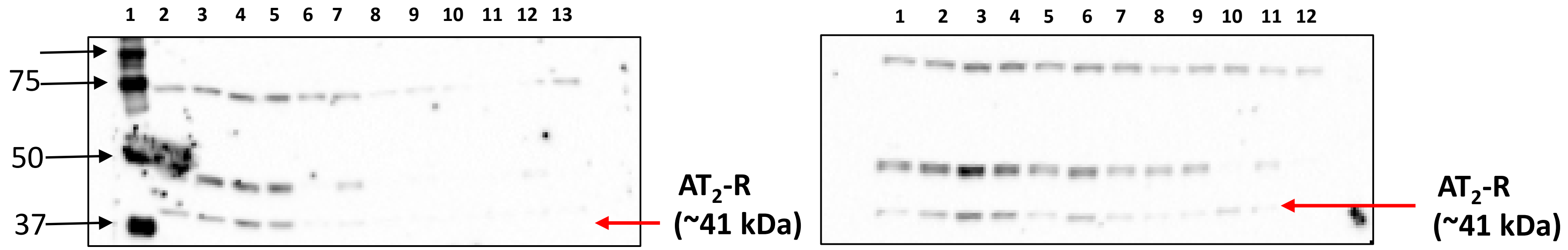

After gel -> membrane transfer, membrane was cut in half. Standard seen on the left picture applies to both.

NTS WKY (1-6), **NTS** SHR (7-12)

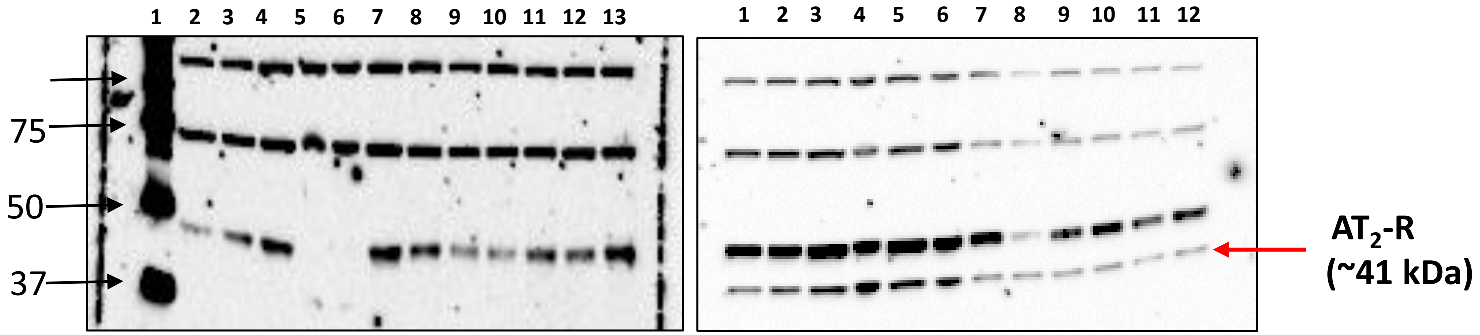

After gel -> membrane transfer, membrane was cut in half in order to use two different antibodies (CB_1-R and AT_2-R). Standard seen on the left picture applies to both.

Figure S1b. Uncropped Western blots shown in Figure 7b.

<u>Mas-R</u>

From the left (rows):

standard (1), **PVN** WKY (2-7), **PVN** SHR (8-13), **RVLM** WKY (14-20), **RVLM** SHR (21-26)

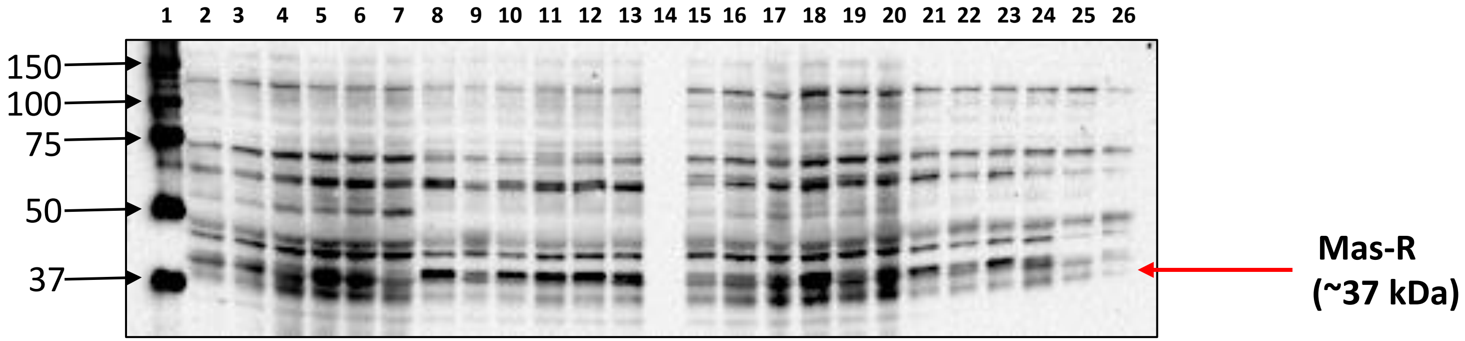

standard (1), **NTS** WKY (2-7), **NTS** SHR (8-13),

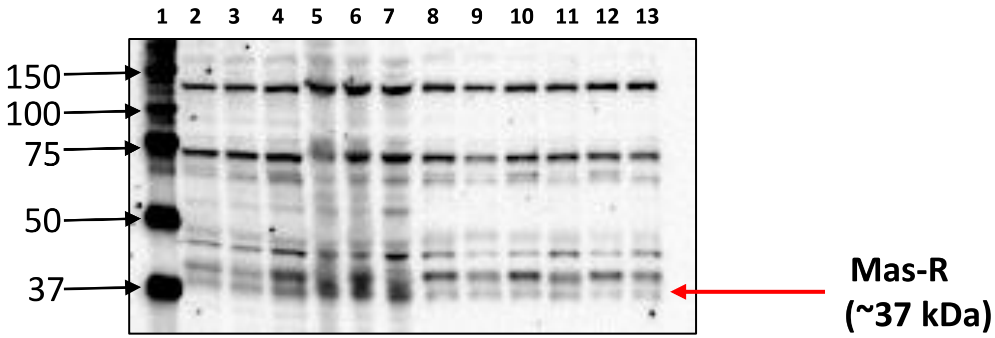

Figure S1c. Uncropped Western blots shown in Figure 7c.
